# Tuberculose disséminée révélée par une localisation épididymaire chez un patient immunocompétent: à propos d’un cas

**DOI:** 10.11604/pamj.2024.48.2.42965

**Published:** 2024-05-02

**Authors:** Rebeh Bougossa, Fatma Larbi Ammari, Asma Ben Mabrouk, Rabeb Jouirou, Sondess Arfa, Jihene Chelli

**Affiliations:** 1 Service d’Endocrinologie et de Médecine Interne, Centre Hospitalier Universitaire de Taher Sfar Mahdia, Mahdia, Tunisie

**Keywords:** Tuberculose disséminée, tuberculose épididymaire, tuberculome, immunocompétence, cas clinique, Disseminated tuberculosis, epididymal tuberculosis, tuberculoma, immunocompetence, case report

## Abstract

La tuberculose épididymaire est rare et pose souvent de problème diagnostique. Elle peut être révélatrice d'une forme disséminée de l'infection, qui est le cas de notre observation. Un jeune homme de 19 ans, sans antécédents notables, était hospitalisé pour une grosse bourse gauche douloureuse évoluant depuis 8 mois. Il avait bénéficié d'une orchidectomie dont l'examen anatomopathologique était en faveur d'une tuberculose épididymaire. Un bilan radiologique avait montré d'autres localisations de l'infection: ganglionnaire, pulmonaire, pariétale et ostéo-articulaire. Un traitement antituberculeux lui était instauré. Cependant, au 4^e^ mois du traitement, le patient avait présenté des crises convulsives. Une IRM cérébrale lui était pratiquée concluant à des tuberculomes cérébraux. Le traitement antituberculeux était poursuivi associé à un anticonvulsivant avec une bonne évolution clinico-radiologique. L'originalité de notre observation réside sur le mode de révélation d'une tuberculose disséminée paucisymptomatique, par une localisation épididymaire, chez un sujet immunocompétent.

## Abstract (en)

Epididymal tuberculosis is rare and often presents diagnostic difficulties. It may be indicative of a disseminated form of the infection, which is the case of our patient. A 19-year-old man, with no past medical history, was admitted for a swollen painful left scrotum that had been evolving for 8 months. He had undergone an orchiectomy and the anatomopathological examination was consistent with epididymal tuberculosis. The radiological investigations had revealed other localizations of the infection: lymphatic, pulmonary, parietal and osteoarticular tuberculosis. Anti-tuberculosis therapy was introduced. However, in the 4^th^ month of treatment, the patient developed seizures. A cerebral magnetic resonance imaging was practiced, concluding to cerebral tuberculomas. Anti-tuberculosis treatment was continued associated to an anticonvulsant with a favourable outcome. The originality of our observation resides in the mode of revelation of a disseminated paucisymptomatic tuberculosis, by an epididymal localization, in an immunocompetent patient.

## Introduction

Malgré les progrès incontestables que l´humanité a connus grâce à la recherche scientifique; la tuberculose demeure encore un problème majeur de santé publique mondiale. La situation est surtout plus précaire dans les pays en voie de développement y compris la Tunisie où l´incidence de la tuberculose est de 34/100.000 habitants [1]. Cette infection atteint principalement les poumons. La localisation épididymaire est rare et peut être révélatrice d´une forme disséminée. Du fait de sa symptomatologie non spécifique; l´incidence exacte de la tuberculose disséminée n´est pas encore connue et son diagnostic est retenu dans 33% à 80% des cas en post mortem [2]. Elle survient souvent chez les sujets immunodéprimés, surtout les patients vivants avec le virus d´immunodéficience humaine (VIH), mais elle peut se voir aussi chez les personnes immunocompétentes. Nous rapportons une observation originale de tuberculose disséminée révélée par une localisation épididymaire chez un patient immunocompétent.

## Patient et observation

**Informations relatives au patient**: un jeune homme de 19 ans, sans antécédents notables, était admis à notre service pour une grosse bourse gauche douloureuse évoluant depuis 8 mois, non améliorée par la prise des antibiotiques visant des germes pyogènes. Le patient rapportait, depuis le début des symptômes, une fébricule intermittente, des sueurs nocturnes, une anorexie et un amaigrissement non chiffré. Il n´avait pas de toux ni des expectorations ni d´hémoptysie. Pas d´histoire de tuberculose dans ses antécédents ni dans son entourage. Il était vacciné contre la tuberculose à sa naissance.

**Résultats cliniques**: l´examen physique révélait une grosse bourse gauche douloureuse légèrement chaude et deux formations sous cutanées allongées, mal limitées, fermes et douloureuses, siégeant à la paroi thoracique antérieure et mesurant 3 à 4 cm de long. Il n´y avait pas des râles à l´auscultation pulmonaire. Le reste de l´examen était normal. Les examens biologiques avaient montré une protéine c-réactive élevée à 90 mg/dl. La numération formule sanguine, la créatininémie, la glycémie et le bilan hépatique étaient dans les limites de la normale. La radiographie standard du thorax avait montré des infiltrats réticulo-nodulaires aux deux champs pulmonaires.

**Figure 1:**
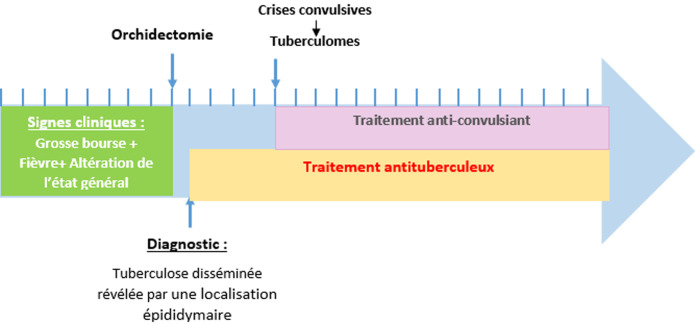
chronologie des signes cliniques et des interventions

**Chronologie**: (Figure 1).

**Figure 2:**
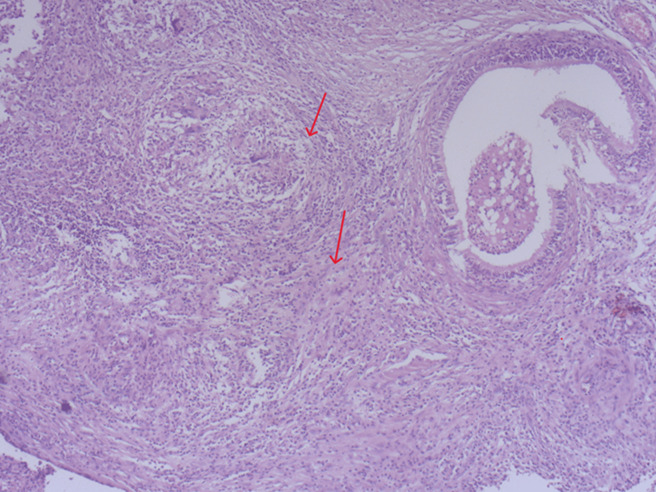
épididymite granulomateuse épithéloide et nécrosante

**Figure 3:**
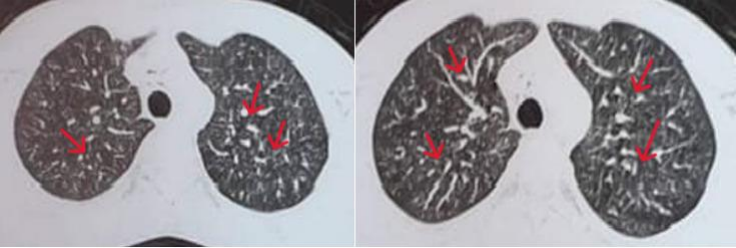
miliaire tuberculeuse à la tomodensitométrie

**Démarche diagnostique**: devant un aspect échographique en faveur d´une tumeur épididymaire, le patient avait bénéficié d´une orchidectomie gauche. Cependant, l´examen anatomopathologique de la pièce opératoire avait montré une épididymite chronique granulomateuse épithéloide nécrosante évoquant une tuberculose épididymaire active intéressant le corps et la queue de l´épididyme et épargnant la tête et le testicule (Figure 2). L´intradermo-réaction à la tuberculine était positive. Les recherches des bacilles acido-alcoolo-résistants (BAAR) dans les crachats et les urines 3 jours de suite étaient négatives à l´examen direct et à la culture. Les sérologies de: virus d´immunodéficience humaine (VIH), virus de l´hépatite B (VHB), virus de l´hépatite C (VHC) et Wright étaient aussi négatives. A la recherche d´autres localisations tuberculeuses, une tomodensitométrie thoraco-abdomino-pelvienne lui était pratiquée objectivant une miliaire tuberculeuse (Figure 3), des adénopathies nécrotiques coelio-mésentériques, deux collections pariétales thoraciques (au dépend des muscles obliques externes fusiformes mesurant 33×10mmm à droite et 45×10 mm à gauche) et une ostéolyse focale de D11 et L5 évoquant une spondylodiscite débutante. L´imagerie par résonnance magnétique (IRM) médullaire avait confirmé l´existence d´une spondylodiscite au niveau de D7, D11, L4 et L5. Le diagnostic d´une tuberculose disséminée avec des localisations épididymaire, pulmonaire, pariétale, ganglionnaire et ostéo-articulaire était retenu.

**Intervention thérapeutique**: une polychimio thérapie antituberculeuse associant l´isoniazide, la rifampicine, l´éthambutol et la pyrazinamide pendant 2 mois, suivie de l´association de l´isoniazide et la rifampicine, était mise en route.

**Figure 4:**
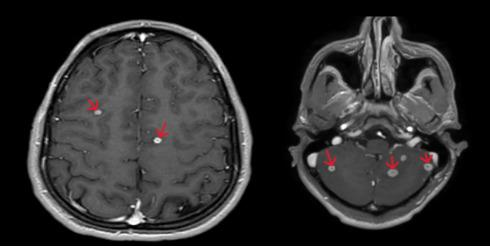
multiples tuberculomes intracrâniens

**Suivi et résultats**: cependant, au 4^e^ mois du traitement, le patient avait présenté des crises convulsives tonico-cloniques généralisées. Une IRM cérébrale lui était pratiquée, objectivant des multiples lésions nodulaires sus et sous tentorielles correspondant à des tuberculomes (Figure 4). Le patient était mis sous un traitement anti-convulsiant et le traitement anti-tuberculeux était poursuivi. L´évolution était favorable avec récupération de l´état général et un gain du poids dès les premiers mois du traitement ainsi qu´une disparition des formations pariétales thoraciques, des adénopathies et de la miliaire tuberculeuse à la tomodensitométrie avec absence de récidive des crises convulsives. Cependant, la diminution lente de la taille des tuberculomes nous obligeait de poursuivre le traitement anti-tuberculeux pendant une longue période. Enfin, le patient était déclaré guéri, sans séquelles, à la fin du 234^e^ mois du traitement avec un recul de 2 ans.

**Perspective du patient**: au début, il n´était pas facile, sur le plan psychologique, que le patient accepte l´orchidectomie qui pourrait être évitée, mais il était, ensuite, soulagé avec le soutien psychologique et l´annonce du diagnostic de sa maladie qui était une infection curable. Il était globalement satisfait de l´évolution favorable d´une infection qui pourrait mettre en jeu son pronostic vital.

**Consentement éclairé du patient**: le patient a donné son consentement.

## Discussion

La tuberculose est une infection bactérienne ayant des implications importantes sur la santé publique. La tuberculose pulmonaire est la présentation clinique la plus commune. La localisation extra-pulmonaire est de fréquence variable, pouvant toucher n´importe quel organe, dont on cite: la tuberculose génitale qui est rare et de diagnostic souvent difficile et tardif notamment en absence de contexte d´infection tuberculeuse. L´atteinte épididymaire, une forme de la tuberculose génitale chez l´homme, est le plus souvent, révélée par une orchi-épididymite résistante au traitement antibiotique [3]. Dans notre cas, la tuberculose épididymaire était initialement diagnostiquée à tort comme une tumeur épididymaire qui constitue le principal diagnostic différentiel de cette rare localisation tuberculeuse. Cette atteinte épididymaire peut être associée à d´autres localisations connues de la maladie ou être isolée ou révélatrice d´une atteinte disséminée. Chez notre patient, la tuberculose disséminée était paucisymptomatique et l´atteinte épididymaire était révélatrice de la maladie. Un faisceau d´arguments épidémio-cliniques et paracliniques nous avait permis de retenir le diagnostic de la tuberculose disséminée: une intradermo-réaction à la tuberculine positive, des lésions anatomopathologiques évocatrices de la tuberculose au niveau de l´épididyme et un aspect radiologique caractéristique d´une miliaire tuberculeuse, d´une spondylodiscite tuberculeuse et des tuberculomes cérébraux chez un patient vivant dans un pays à endémicité tuberculeuse intermédiaire. Les principaux facteurs prédisposant à cette maladie sont: l´infection par le VIH, le diabète, l´alcoolisme, les tumeurs malignes, la cirrhose, l´insuffisance rénale terminale, les immunosuppresseurs, les connectivites… [2,4,5]. Notre patient n´avait aucune de ces pathologies suscitées ainsi que les sérologies des VIH, VHB et VHC étaient négatives. Dans la série de Wang *et al*., 47% patients atteints de tuberculose disséminée étaient immunocompétents [2]. La présentation clinique de cette affection est variable, dépendant des organes atteints, mais les signes généraux comme la fièvre (94%) et l´altération de l´état général (65%) dominaient le tableau clinique selon l´étude de Crump *et al*. [4] et leur présence doit poser le diagnostic de la tuberculose même chez des sujets immunocompétents [6].

Quant à la biologie, elle est de même non spécifique, mais ils en existent des paramètres qui peuvent prédire la forme disséminée lors d´infection tuberculeuse comme: des taux sériques élevés de phosphatases alcalines, de LDH, de calcium (> 2.6 mmol/l), de ferritine (>1000 ug/L) et de Vitamine B12 ainsi qu´une hypoalbuminémie [2,7]. Chez notre patient, les différentes localisations tuberculeuses ont été mises en évidence progressivement grâce aux examens radiologiques, d´où l´intérêt de pratiquer un bilan lésionnel complet devant toute atteinte tuberculeuse. La spondylodiscite tuberculeuse ou mal de Pott est l´atteinte ostéo-articulaire tuberculeuse la plus fréquente, qui touche souvent l´étage thoracique, cependant l´atteinte multi-étagée est assez fréquente [8], ce qui est le cas dans notre observation. L´atteinte tuberculeuse de système nerveux central est rare, mais elle représente une des formes extra-pulmonaires les plus graves, pouvant mettre en jeu le pronostic vital du patient. Le tuberculome intra-crânien est une masse de tissu granulomateux tuberculeux entourée par le tissu cérébral normal avec un œdème péri-lésionnel. Il peut se localiser dans n´importe quelle partie de névraxe cérébral: sus ou sous tentoriel, ou médullaire. Sa confirmation diagnostique se fait normalement par une biopsie stéréotaxique. Cependant des aspects radiologiques caractéristiques, comme le « Target sign », associées à un contexte épidémio-clinique évocateur, permettent souvent de retenir le diagnostic. L´augmentation paradoxale de la taille des tuberculomes cérébraux, au cours des premiers mois du traitement anti-tuberculeux, peut expliquer la survenue des convulsions chez notre patient. En effet, les tuberculomes cérébraux représentent avec les atteintes ganglionnaires et pleurales les manifestations les plus fréquentes des réactions paradoxales au traitement anti-tuberculeux chez le sujet immunocompétent [8]. Cette réaction ne signifie pas un échec thérapeutique et la poursuite du traitement anti-tuberculeux aboutit à la guérison dans la plupart des cas [9]. Hijazi *et al*. ont recommandé de ne pas changer le traitement anti-tuberculeux et d´ajouter la dexaméthasone en intraveineux pendant 4 à 8 semaines lors d´une réponse paradoxale des tuberculomes au traitement anti-tuberculeux [10]. Notre patient n´avait pas reçu de corticothérapie ce qui pouvait expliquer en partie l´amélioration lente de son état.

## Conclusion

L´originalité de notre observation réside dans le mode de révélation d´une tuberculose disséminée, par une localisation épididymaire, chez un sujet immunocompétent, diagnostiquée initialement à tort comme une tumeur épididymaire. Un bilan radiologique exhaustif devant toute localisation tuberculeuse parait nécessaire.
